# Allegheny County Medical Society

**Published:** 1891-02

**Authors:** W. S. Foster

**Affiliations:** President in the Chair


### ALLEGHENY COUNTY MEDICAL SOCIETY.

*Special Meeting, December 16th,* 1890.

W. S. FOSTER, M. D., PRESIDENT IN THE CHAIR.

### ALBUMINURIA AFTER TYPHOID FEVER.

Dr. Batten: A girl eleven years of age convalesced and became apparently well, September 9th, after a malignant attack of typhoid fever. On October 24th she had a shuffling walk and depression of the left shoulder. She also had pain in the abdomen. The following morning I visited her and concluded that the depression of the left shoulder was from irritation of the spine. Upon examination of the urine, I found that it was highly charged with albumen and there were no symptoms of paralysis except the depression of the left shoulder. She had use of her left leg and arm but did not use them as well as she did the right. I put her to bed, cupped her over the back and applied poultices over the abdomen and put her on nitroglycerin. She did not appear to improve under this treatment, and I changed it to iodide of potash in doses of five grains every three hours. Under this treatment the albumen diminished and finally disappeared, and the shoulder took its normal position. On November 30 I discharged her, well. It is the first case of albuminuria I have had following typhoid fever.

### FRACTURE OF THE RADIUS.

Dr. Murdoch: My attention has been called to an article in the *Medical News*, a paper by Dr. Roberts, of Philadelphia, which was read before the Academy of Surgery two weeks ago. The title of the paper is "The uselessness of splints in the treatment of fractures of the lower end of the radius." The paper itself is interesting and the discussion which it elicited also, and as there were many points made by Dr. Roberts which I heartily approve of and which differ from those usually received by the profession, I thing it interesting to revive the old hackneyed subject of fractures of the lower end of the radius. There is no fracture that has been more discussed, there are points not yet settled, and difference of opinion among good surgeons. It is comparatively a few years since this fracture at the lower end of the radius was thought to be a dislocation, and was so regarded by all surgeons not one hundred years ago and always described as a dislocation. Some surgeons contend that the fracture is always caused by cross strain in hyper-extension of the wrist. But so good a surgeon as Dr. Stimson, of New York, argues that it never occurs in that way; that it is generally the result of a compressing force, the shaft of the radius being driven into the fragment of the bone by a downward force. He asserts that in the living body the strain cannot be in such a way as to produce this fracture. The fracture, as you know, occurs about half an inch above the lower extremity of the joint, from three-eights to three-fourths of an inch from the lower extremity of the radius, and the fragment is driven upwards and backwards upon the shaft, and described by all surgeons as the silver-fork fracture. The fragment is driven upon the shaft. The reason so much difference of opinion exists is owing to the fact that different surgeons see cases of this fracture produced by different degrees of violence. As we usually see the fracture, it occurs in old ladies slipping and falling and receiving the weight of the body upon the bones, and the radius gives way by a slight amount of violence; these are the most common cases. But surgeons like Dr. Moore, of Rochester, who have made *post mortem* examinations of patients with fractures of the radius give an entirely different account of it. Dr. Moore relates the case of a patient who fell from a third story window in a lunatic asylum, head foremost, striking on both arms; there was a fracture of the lower end of the radius and a crushing of the lower fragment, also a dislocation of the ulna. In this case the styloid process of the ulna was entangled in the annular ligament.

In the treatment of this fracture at first the pistol-shaped splints were applied for the purpose of abducting the hand toward the ulnar side, and all the splints for a great many years afterward were of that type. The idea being to draw the hand to the ulnar side, supposing that by that movement the lower fragment would be drawn down into place. Then again, Gordon, of Dublin, advised a splint by which the hand was flexed, and Dr. Kearns has a splint of his own, the retroflexed splint, and there are still other varieties. Now, here comes a good surgeon who asserts that all these splints are useless. I am inclined to think, looking back upon several of my own cases, that he is practically right. If we recollect how loosely the carpal bones are connected with the radius, and the great amount of motion that the hand normally has, in flexion and extension and lateral movements, the idea that one can influence the position of the lower fragment by altering the position of the hand seems absurd. The hand was flexed toward the ulnar side because of the mistaken impression that there was a close connection between the ulnar and the cuneiform bone, and that by pulling the hand in that direction, the lower fragment would be pulled into place. Owing to this loose connection between all the carpal bones it is impossible to influence the lower fragment by any position of the hand. This is true whether it is flexed or turned to the radial or ulnar side, or whether you retroflex it after Dr. Kearns. You do not by any of these methods influence the lower fragment, which is only three-fourths of an inch long. Besides if you put pressure enough upon the lower fragment to influence it by any splint you are very likely to stop the circulation in the hand. Prof. Hamilton has related five cases of gangrene of the hand by tight splints in this connection. The vessels are so easily compressed that the circulation is readily cut off, and any pressure by splints that would be likely to influence the lower fragment would be likely to arrest the circulation in the hand. The chief deformity after this fracture is the stiffness of the fingers. What is the cause of this stiffness? It is asserted usually to be that the fingers are kept still for so long a time. But this is an error. The stiffness in the fingers and wrist is owing to the inflammation in the joint itself, and in the sheaths of the tendons. What is it that causes the inflammation of the joint and in the sheaths? It is, no doubt, largely due to the proximity of the fracture, but is it not probable that a good deal of it may be caused by this strong pressure made by the splints? The fact is, gentlemen, I have seen, as Dr. Roberts says he has seen, cases where splints were put on without any reduction of the fracture whatever. The idea is too prevalent that a fracture can be treated simply by a splint. A fracture of the radius usually occurs in a transverse direction, and if the fracture is reduced, *i. e.*, the fragment put back where it belongs, nine times out of ten it remains there without a splint. I say this is usually the case. This fracture differs from other fractures. Usually we can reduce a fracture easily, the difficulty being its retention. In this fracture the difficulty is in reduction; retention is easy. When reduction is accomplished all that can be done is done. The fragments fit into each other, there is no muscular action to displace them. Generally speaking, you have done all you can do when you have reduced the fracture. This is so when the case occurs through a minimum amount of violence. In these fractures in the lower end of the radius where the shaft of the bone is driven violently against its lower extremity, and the lower fragment is split and crushed, pulverized as it were, you will not be able with any splint to prevent a deformity. A portion of the bone has been lost. I do not go so far as to say splints are useless. But I assert that so far as the replacement of the fragments is concerned, the reduction is the principal thing to attend to. I have seen within a week a patient come to my office with a Colles fracture two weeks old, with the fragment out of place as much as it was at the time of the accident, and was able, with a good deal of force, to reduce it. The man had on a Gordon splint. I have within three months seen five or six cases of this fracture. I believe that the kind of splint is not important. I think it well to put on some kind of a splint. A fracture so near the joint must necessarily be painful to the patient. I think for comfort and quiet, it is well to put on some kind of a splint to abolish motion of the joint, but only for that purpose. If the fracture is reduced, it will remain reduced. If the fragment is crushed, then no splint will lessen deformity. I also believe that one of the principal things to be observed in the treatment is, that no dress ing shall be put on so tightly as to press upon the sheaths of the tendons, for this contributes to the stiffness in the joints. Passive motion, as I have already said before this Society, I do not believe in. I believe the fracture should be kept quiet until there is some union between the fragments. In the treatment of this fracture the important thing is to reduce it.

Dr. Thomas : I would like the doctor to tell us whether he meets with uniformly good results or poor results. I have seen a number of cases of Colles' fracture, and I must say my results have not been perfect. The doctor states the important thing to do is to reduce the fracture. If you have done that you have done all that is necessary. Now I take issue with the doctor there. I do not believe the principal thing is to reduce the fracture. The difficulty in the fracture is the question of deformity and the amount of dislocation of the ulna. If I get a case of fracture with dislocation of the ulna I know I am going to have trouble. If the bone is entangled in the annular ligament, and if I can get that bone reduced, I do not care so much for the fracture. I had a case of that kind within the last three months in a woman about sixty years of age—dislocation of the ulna, and I thought I was going to have a very good case, and I think I will yet. I do not believe in any special splint. If you can succeed in reducing the ulna, any kind of splint will keep it in place, and the lighter the better. In this case I applied anterior and posterior splints and bound very lightly and only allowed them to remain on the hand ten days. Then I took off one and allowed the other to remain. At the end of four weeks I took off the other splint, and I observed what I did not observe when I dressed the hand, there was much more deformity at the wrist joint than at the back of the hand. In other words, inflammation had been going on in the coverings of the tendons and the tendons themselves. I expect this is about as good a result as we can generally get in a Colles fracture. The doctor says the splint produces this inflammation. I believe it is the original injury. I think the trouble in the tendons is altogether from the injuries received at the time. If some one could tell me how to reduce the ulna I would feel that I could treat these cases very well.

Dr. Brashear, of Cleveland: This subject of fracture is always one of much interest to me, and it seems to me we are slow in learning the best and most successful methods of treating this most common fracture. When I began the practice of setting bones, the first splints I used were two splints, cigar-box splints, and when the ulna was not displaced, my success was satisfactory. If it was displaced, I suppose my success was about equal to that of my contemporaries. I used, I think, nearly all the splints up to the time when I ceased the use of the splint for a fracture. I have used the splint of Dr. Geo. F. Shrady, of New York, which is a simple one, but its puts the hand in a cramped shape, painfully so. I still have a splint of that kind and would like to put it in some museum. Then I used the pistol-shaped splint which I made myself; and I have used the splint of a former member of your society, George McCook, of this city. Perhaps about the year 1868 I ceased using splints for this fracture. I have not used a splint since, and have no intention of using a splint for Colles' fracture, and will not use one unless I see some splint that will produce better results than the method I employ. The gentleman who introduced this subject poke of the case Dr. Moore had the opportunity of studying, that of the patient who fell from a third story window upon both hands, and had a double fracture. The *post mortem* was made very soon after death, and the details of that case were very intelligently written out and published. Shortly after—I had occasion to make a *post mortem* of a fracture. I desired to see whether the short fragment was in relation to the hand as asserted by Dr. Moore. An old lady fell backwards down a step twelve inches high, and in falling put out one hand to save herself and was picked up dead, carried into the house and I was sent for. We arranged for a *post mortem.* I was anxious to see this fracture, because I was then engaged with the dressing of Dr. Moore, and this *post mortem* demonstrated that the short fragment was turned sidewise. I found it exactly as Dr. Moore said it would be. The study was a very interesting one. I saw the mechanism of the fracture, if the ulna can be replaced, and since I have been following the method of Moore I have not found one which I could not restore. After that is properly restored the rest of the treatment is simple ; it is very easy and no splint is needed. Prof. Moore puts a little roller bandage, the diameter of which will not exceed the thickness of the wrist and about an inch wide on the palmar surface and just back of the wrist joint, allowing the weight of the hand to make the extension; this is surrounded with a bit of adhesive plaster as wide as the the bandage, put on moderately tight. I cannot say too much in favor of Dr. Moore's method, and that was published a good many years ago. Why we do not all use it I do not know. I should be very sorry to have a roll-call of my patients treated with the pistol splint. I think that is the worst of all ; it is the worst a man can do. Better do nothing.

Dr. Daly: This subject at once calls to my mind the experience I had with our friend Dr. Brashear, sixteen or seventeen years ago. I was present when he dressed the fracture in the manner which he has described. I watched the case with considerable interest, and his results, as I remember them, were satisfactory. It is well known to all gentlemen who have treated fractures that you cannot lay down any hard, fast rules for all cases. It is true I have not treated a fractured bone for many years, at least no other fracture than fracture of the nose. But for many years previous to the many years I speak of I treated a great many, when in general practice, and as I recall the cases which I had, I have no great sense of pride in my results. I believe that at one time I gave considerable amount of study to Colles' fracture, made some dissections, and the outcome of that study was a paper which I read before the Mott Medical Club. As I remember, the position I took then, if I were engaged in the practice of surgery and treatment of fractures, I would probably take now. We, as surgeons and physicians, ought, upon general principles, to combat whatever evil may result from accident or disease. Now, we all know the greatest evil of a fracture is the transferring of the axis of the hand to the radial side. If you will examine a given number of fractures in which there have been bad results, you will find a prominence of the styloid process of ulna, as I say, the transferring or bringing over the axis of the hand to the radial side. After seeing our friend Dr. Brashear dress this case of fracture, I am not quite sure that I did not call him in consultation with my next case. I cannot remember particularly, but I have a general recollection that the cases treated by the method he speaks of were followed by as good results as the cases wherein the method I was so fond of using was employed. This was the method proposed by Dr. Walter, of Pittsburgh. The results in treating Colles' fracture by the Walter splint are usually good. So, to sum up, it is about like this: While Dr. Murdoch very justly says a very important point is to reduce your fracture—that, of course, we know is an important point with all fractures—there is another important point which appeals to every one who has had experience. That is to use measures as simple as possible to retain your dislocated or relocated bone in apposition, and we who have had experience know that it is not easy to retain the dislocated and relocated bones in proper apposition. The most important point is to retain your bones in apposition. Do not pin your faith too closely to the non-splint treatment, and do not pin your faith too closely to the splint treatment. I am very much pleased, indeed, to have had this subject brought up.

Dr. Batten: I am of the opinion of Dr. Murdoch, that if you once reduce the fracture properly it makes little difference what sort of a splint you use. I have used all kinds of splints; I have made splints of my own, pistol shingles with roller bandage on the ends, and had good results with them. But I have always been very particular to reduce the fracture properly. The first time my notice was drawn to Dr. Moore's treatment with a simple plaster, the patient was under the care of Dr. Brashear, and I do not know whether it was the same patient Dr. Daly speaks of, but my recollection is that it was. I believe that some patients are more susceptible of inflammation than others. I have treated cases that would have no difficulty with inflammation, and then, again, others where there was great trouble. The last case I treated was a woman about forty years old. She gave me a great deal of trouble for about six months, but at the end of that time her arm became very useful, and there was not any deformity.

Dr. McCann: This subject will always be a source of discussion. There are cases which will recover without deformity and others which will not. The fractures in the vicinity of the wrist joint are really three. First, Barton's fracture, which simply consists of a sliding off of the portion of the radius directly into the joint, a fracture which, although Barton saw and described, he never saw the pathological condition of; it was afterward seen by other persons. Another fracture is the fracture which occurs from a half to three-fourths of an inch above the wrist joint, and there is still another fracture, Robert Smith's fracture, an inch and a half above the wrist joint. These fractures are very commonly described or classified together, but anybody who will take the trouble to look at the anatomical construction of the parts, will readily see that there must be a very great difference in the treatment and in the results of that treatment. In the first place, you have a fracture involving the wrist joint very often with rupture of the annular ligament, and consequently a fracture which, unless carefully reduced, must necessarily be followed by deformity. Now, that is not so likely to occur with the fractures three-fourths of an inch above the wrist joint which do not involve the joint at all, although they may be attended by displacement. In Smith's fracture, an inch and a half above the joint, these conditions are not likely to be present, consequently in the cases which are treated as Colles' fracture, which are really Smith's fracture, the results must necessarily, with any kind of careful treatment, be good. With Colles' fracture, the results ought to be good if carefully treated. With Barton's fracture, involving the joint with displacement of the ulna and comminution of the fragment, we have conditions which are likely to be followed by deformity no matter what method of treatment is used. Now, I have treated a few of these cases. When I have a fracture in the vicinity of the wrist joint, the first thing I do is to reduce the fracture if I can. If you have them there, it is not very difficult to retain them. The old practice initiated by Sayre of the treatment of all fractures consisted in extension and retention, no matter what apparatus you use, so that it accomplished the object. Prof. Moore claims that he treats his cases simply by adjusting the fragment by drawing the hand powerfully to the radial side and simply putting his light compress below, holding it in position by adhesive plaster, and I have no doubt he obtains good results. I think that in the treatment of this fracture a great deal depends upon where it is located, whether it involves the joint, or an inch and a half above the joint.

Dr. Kearns: There is nothing I can say to throw any light on the origin of my splint. To be brief, I should say this splint is the evolution of experience. I found that when I treated fractures of the forearm that there would often be swelling for months afterward, interfering with the daily employment of the injured person. My desire to remedy this difficulty was the cause of my studying this matter. In presenting these splints to the New York City Hospital, they said that the principle I spoke of was the principle they had been trying for years, but failed to rightly employ. Take one of the arms not broken, put it in a straight splint, keep it there for three or four weeks, there will be a stiff, immovable arm. Now, again, another qualifying cause of this. The cases I had were nearly all hard-working men, anxious to return to their work as soon as possible. They could not appreciate fine-spun theories, but did want quick relief, and if I could not give it they were not backward about going to another physician. To meet their cases I devised the splint of which I speak, and have had good success in its use.

Dr. Murdoch: In the first place, I want to say to Dr. Thomas, I am rather proud of my results. I have had good success, but I will say that I have not always treated them on the principle that I have given here to-night. These are nearly new ideas with me. I have been in the habit of treating like others. I find from the discussion which has taken place that there is the same diversity of opinion among the members here, and for the same cause that I referred to in my opening remarks. Those surgeons who have seen only the minor form of the injury take one view of the case, whereas those who have seen the graver injury, like Dr. Moore's case, take another view, and advocate different treatment. When I get a case of fracture of the radius accompanied by dislocation of the ulna, I will treat it as Dr. Moore did, but the idea that all the fractures of the radius are accompanied by dislocation of the ulna has been abundantly disproved by dissections immediately after the injury.

I will now answer the question asked by my distinguished friend D. Brashear, why all fractures of the radius are not treated in that way. It is because it has been proven by abundant dissection that all of the fractures of the radius are not accompanied by dislocation of the ulna, and that is a sufficient answer to Dr. Thomas' argument that there is a dislocation of the ulna in the majority of fractures of the radius. Dr. Brashear is the only surgeon of eminence that I know who now treats all fractures of the radius after the manner of Moore. I am familiar with Moore's treatment, I know his method. He told it in 1868—twenty-two years ago. It has not been adopted by the profession. The simple fact that it has not been adopted by the profession, after having been presented by such a distinguished man as Dr. Moore, shows that the profession does not regard that as the usual injury. Dr. Pilcher, of Brooklyn, made an elaborate study of this subject and asserts the reason of the deformity is the prevention of reduction by the un-torn periostium. But the point I want to emphasize is that the reduction of the fracture is the principle thing to attend to in the simpler forms of injury where there is a transverse fracture. If the fracture is reduced, it will remain reduced. Dr. Daly has well said that the proper treatment of all fractures is to reduce them and then retain them in apposition. In fracture of the thigh, the great difficulty is to retain the fragments in position, the extension and counter-extension must be kept up for a long period. In this fracture at the lower end of the radius there is no muscular action to displace the fragments after reduction. The interlocking of the fragments is sufficient to retain them in position, and where you have the simpler fracture of the radius, you will have very little deformity.

Dr. Batten: Will Dr. Murdoch please tell us his method of reducing this fracture ?

Dr. Murdoch: Certainly. The surgeon shall sit down by the side of his patient, and taking the forearm upon his knee, grasp it so that the fingers of both hands will rest upon the palmar prominence, and the thumbs upon the dorsal one, making steady and firm pressure *upon the lower fragment* in a direction downward and forward, until it is pressed into place.

